# Anti-Biofilm Activity of a Long-Chain Fatty Aldehyde from Antarctic *Pseudoalteromonas haloplanktis* TAC125 against *Staphylococcus epidermidis* Biofilm

**DOI:** 10.3389/fcimb.2017.00046

**Published:** 2017-02-23

**Authors:** Angela Casillo, Rosanna Papa, Annarita Ricciardelli, Filomena Sannino, Marcello Ziaco, Marco Tilotta, Laura Selan, Gennaro Marino, Maria M. Corsaro, Maria L. Tutino, Marco Artini, Ermenegilda Parrilli

**Affiliations:** ^1^Department of Chemical Sciences, Federico II University, Complesso Universitario Monte Sant'AngeloNaples, Italy; ^2^Department of Public Health and Infectious Diseases, Sapienza UniversityRome, Italy

**Keywords:** anti-biofilm, *Staphylococcus epidermidis*, long fatty acid aldehyde, quorum sensing, *Pseudoalteromonas haloplanktis* TAC125

## Abstract

*Staphylococcus epidermidis* is a harmless human skin colonizer responsible for ~20% of orthopedic device-related infections due to its capability to form biofilm. Nowadays there is an interest in the development of anti-biofilm molecules. Marine bacteria represent a still underexploited source of biodiversity able to synthesize a broad range of bioactive compounds, including anti-biofilm molecules. Previous results have demonstrated that the culture supernatant of Antarctic marine bacterium *Pseudoalteromonas haloplanktis* TAC125 impairs the formation of *S. epidermidis* biofilm. Further, evidence supports the hydrophobic nature of the active molecule, which has been suggested to act as a signal molecule. In this paper we describe an efficient activity-guided purification protocol which allowed us to purify this anti-biofilm molecule and structurally characterize it by NMR and mass spectrometry analyses. Our results demonstrate that the anti-biofilm molecule is pentadecanal, a long-chain fatty aldehyde, whose anti-*S. epidermidis* biofilm activity has been assessed using both static and dynamic biofilm assays. The specificity of its action on *S. epidermidis* biofilm has been demonstrated by testing chemical analogs of pentadecanal differing either in the length of the aliphatic chain or in their functional group properties. Further, indications of the mode of action of pentadecanal have been collected by studying the bioluminescence of a *Vibrio harveyi* reporter strain for the detection of autoinducer AI-2 like activities. The data collected suggest that pentadecanal acts as an AI-2 signal. Moreover, the aldehyde metabolic role and synthesis in the Antarctic source strain has been investigated. To the best of our knowledge, this is the first report on the identification of an anti-biofilm molecule form from cold-adapted bacteria and on the action of a long-chain fatty aldehyde acting as an anti-biofilm molecule against *S. epidermidis*.

## Introduction

*Staphylococcus epidermidis* (*S. epidermidis*) is now being recognized as an important opportunistic pathogen that can cause significant problems when breaching the epithelial barrier, especially during the biofilm-associated infection of indwelling medical devices (Otto, 2008; Arciola et al., 2012). Most diseases caused by *S. epidermidis* are of a chronic character and occur as device-related infections (such as intravascular catheter or prosthetic joint infections) and/or their complications (Dohar et al., 2009; Artini et al., 2013). Implantations of medical devices are steadily increasing and thus heightening the relevance of *S. epidermidis* as a human pathogen. The ability of *S. epidermidis* to adhere on both eukaryotic cells and abiotic surfaces and to form biofilm is an essential virulence factor that contributes to the chronicization of infections particularly difficult to eradicate. Biofilms are sticky, surface-attached agglomerations of bacteria that are embedded in an extracellular matrix and provide protection for single cells from antibiotics and mechanisms of host defense (Epstein et al., 2012).

*S. epidermidis* infections are estimated to reach 250,000 cases per year in the USA with a mortality rate of up to 25%. The interest in the development of innovative approaches for the prevention and treatment of staphylococcal adhesion and biofilm formation capabilities has therefore increased. A viable approach should target the staphylococcal adhesive properties without affecting the bacterial viability in order to avoid the rapid appearance of escape mutants (Papa et al., 2015).

From another point of view, biofilm could be considered as a source of novel drugs. Indeed, the specific environmental conditions prevailing within biofilms may induce a profound genetic and metabolic rewiring of biofilm-dwelling bacteria and therefore may allow the production of metabolites different from those obtained in the planktonic condition. Indeed, many bacterial biofilms secrete molecules such as quorum sensing signals (Ni et al., 2009), surfactants (Kiran et al., 2010), enzymes (Kaplan, 2010), and polysaccharides (Valle et al., 2006; Qin et al., 2009) that act by regulating the biofilm architecture or mediating the release of cells from biofilms during the dispersal stage of the biofilm life cycle (Kaplan, 2010). Furthermore, the production of extracellular molecules that degrade adhesive components in the biofilm matrix is a basic mechanism used in the biological competition between phylogenetically different bacteria (Kaplan, 2010).

Starting from the idea that the production of an anti-biofilm compound might result from the selective pressure exerted on bacteria living in oligotrophic and extreme environments like Antarctica, cold-adapted marine bacteria have been investigated as a possible source of anti-biofilm molecules. Previous papers (Klein et al., 2011; Papa et al., 2013b, 2015; Parrilli et al., 2015, 2016; Sun et al., 2015) have confirmed that cold adapted bacteria represent an untapped reservoir of biodiversity able to synthesize a broad range of potentially valuable bioactive compounds (Klein et al., 2011), including anti-biofilm molecules (Papa et al., 2013b; Parrilli et al., 2015, 2016).

In particular, the Antarctic bacterium *Pseudoalteromonas haloplanktis* TAC125 (*P. haloplanktis* TAC125), when grown with a sessile life-style, proved to be able to secrete an anti-biofilm molecule capable of inhibiting *S. epidermidis* biofilm formation (Papa et al., 2013b; Parrilli et al., 2015, 2016). This anti-biofilm compound impairs biofilm development and disaggregates the mature biofilm of *S. epidermidis*, even in dynamic conditions, without affecting the bacterial viability, showing that its action is specifically directed against biofilm (Papa et al., 2013b; Parrilli et al., 2015, 2016).

In this research study the anti-biofilm molecule produced by *P. haloplanktis* TAC125 has been purified and characterized. In detail, a *P. haloplanktis* TAC125 biofilm cultivation in automatic bioreactor (Parrilli et al., 2016) has been used to obtain a cell-free supernatant in a sufficient amount to purify and characterize the anti-biofilm molecule. A suitable purification protocol was developed using an activity-guided fractionation strategy. The structure of the purified molecule, obtained by NMR and mass spectrometry, corresponded to pentadecanal, a long chain fatty aldehyde. Several experiments were performed to assess the chemical features responsible for its activity, by testing chemical analogs differing in the length of the aliphatic chain and in their functional group properties. Therefore, the anti-biofilm activity of different long chain alcohols and aldehydes on *S. epidermidis* biofilm was evaluated. As for the anti-biofilm mode of action, the results reported demonstrated that the long-chain fatty aldehyde works as an AI-2 signal, suggesting that it may interfere with the *S. epidermidis* quorum sensing system. Moreover, in this paper we have investigated the role of pentadecanal in the metabolism of the source strain, in order to clarify if the molecule regulates biofilm development also in the Antarctic bacterium.

## Materials and methods

### Bacterial strains and culture conditions

Bacterial strains used in this work were: *S. epidermidis* O-47 isolated from clinical septic arthritis and kindly provided by Prof. Gotz (Heilmann et al., 1996); *S. epidermidis* RP62A reference strain isolated from infected catheter (ATCC collection no. 35984); *P. haloplanktis* TAC125 (Médigue et al., 2005) collected in 1992 from seawater near French Antarctic Station Dumont d'Urville. Bacteria were grown in Brain Heart Infusion broth (BHI, Oxoid, UK) and in Tryptic Soy Broth (TSB, Oxoid, UK). Biofilm formation was assessed in static condition while planktonic cultures were performed under vigorous agitation (180 rpm).

*Vibrio harveyi* BB170 (luxN::Tn5kan) (ATCC®BAA-1117™) was grown as suggested by suppliers, cultivated in the Autoinducer Bioassay (AB) Medium (Taga, 2005) and incubated aerobically on a rotary shaker at 30°C under vigorous agitation (180 rpm).

All strains were maintained at −80°C in cryovials with 15% of glycerol.

### Bioluminescence assay

To screen the ability of the compounds to interfere with QS, the test products were serially diluted in the AB medium using sterile, black, clear-bottom 96-well microtiter plates (Greiner Bio-one #655090) as assay platform. An overnight culture of *V. harveyi* BB170 was diluted 1:100 in fresh AB medium and incubated at 30°C up to a visible increase in basal bioluminescence. 100 μl of the diluted culture were dispensed inside each well in the microtiter plate starting from a cellular density of about 0.05 (OD)_600nm_; 100 μl of opportune dilutions of pentadecanal in AB medium were added into each well. Luminescence and (OD)_600nm_ were monitored every 15 min over 18 h with a high-performance multimode plate reader (GloMax® Discover System, Promega) in order to correlate the effects of the products on both the growth and bioluminescence kinetics. Data are reported as light units (LU).

### Large scale biofilm cultivation of *P. haloplanktis* TAC125 in automatic bioreactor for the anti-biofilm compound/s production

*P. haloplanktis* TAC125 bacterial culture was grown in BHI medium in a Stirred Tank Reactor 3 L fermenter (Applikon) connected to an ADI-1030 Bio Controller (Applikon) with a working volume of 1 L. The bioreactor was equipped with the standard pH-, pO_2_-, level-, and temperature sensors for the bioprocess monitoring. To allow the biofilm formation, autoclaved solid polystyrene supports were added into the bioreactor (33 supports in 1 L). The culture was carried out at 15°C for 48 h, or at 4°C for 96 h, in aerobic conditions using airflow of 6 L/h, without stirring. After incubation, supports were removed from the supernatant and the adherent cells were recovered by sonication as previously described (Parrilli et al., 2016). In parallel the supernatant was recovered and further separated from cells by centrifugation at 13,000 rpm, sterilized by filtration through membranes with a pore diameter of 0.22 μm and stored at 4°C until use.

### *P. haloplanktis* TAC125 growth in aerobiosis and microaerobiosis

Batch cultivations were performed in a computer-controlled bioreactor (Sixfors System, Infors) equipped with control units for pH and temperature, an oxygen sensing electrode measuring the solution oxygen pressure, and mechanical stirring (200 rpm) at 4°C. Each fermentation unit, filled with medium and sterilized by autoclaving, was equilibrated at process temperature. In order to allow oxygenation of the medium, the stirring speed was set up at the maximum value to be used during the fermentation, and then sterile air supply was switched on. The system was left to stabilize for at least 30 min to guarantee the saturation of the medium with air. In these conditions 100% of measured oxygen pressure was assigned. The zero-point set calibration was performed by saturating the medium with sterile nitrogen gas. Under aerobiosis conditions, measured oxygen pressure was maintained always above 20% by modifying stirring speed and aeration rate. In microaerobiosis (measured oxygen pressure always below 5% saturation) air supply was stopped after inoculum. For each strain, the growth kinetics were followed in triplicate in at least two independent experiments.

### Molecular methods and reagents suppliers

Standard methods were employed for DNA manipulation and isolation, amplification by PCR, and DNA sequencing. Restriction enzymes, T4 DNA ligase, alkaline phosphatase, T4 polynucleotide kinase, Klenow fragment, Taq DNA polymerase were supplied from Boehringer-Roche, Amersham-Pharmacia Biotech, Promega, and New England Biolabs. DNA fragment purification was carried out with the QUIAEX II kit from Qiagen GmbH.

### Vector pVSb0219 and *P. haloplanktis* TAC125-b0219 mutant construction

PCR was employed to amplify a DNA fragment of PSHAb0219 gene. *P. haloplanktis* TAC125 genomic DNA was used as PCR template and two primers were designed to amplify a 275 bp-long region of the PHSAb0219 gene and to introduce an *Eco*RI and a *Sph*I site (Oligo b0219*Eco*RIfw 5′-CTATGAATTCAAGAAGATATTTACGAGC-3′ and Oligo b0219*Sph*Irv 5′-AATACCCGCATGCCGTTGGTGCC-3′). The amplified DNA fragment was digested by *Eco*RI and *Sph*I, and inserted into the pVS plasmid (Giuliani et al., 2012) corresponding site, thus obtaining the pVSb0219 vector. The vector was mobilized by intergeneric conjugation into *P. haloplanktis* TAC125 cells and insertion mutants were screened on plates at 4°C containing carbenicillin (30 μg/ml) as selection agent.

### RNA preparation and RT-PCR

Total RNA was isolated from 500 μl aliquots of *P. haloplanktis* TAC125 cells recovered from both planktonic growth and, after sonication of supports, biofilm growth as reported in literature (Rippa et al., 2012). RNA was reverse transcribed using SuperScript II RNase H- reverse transcriptase (Invitrogen) according to the manufacturer's instructions. PHSAb0219 was reverse transcribed starting from total RNA (~5 μg) using as primer-specific oligonucleotide designed on the 3′ region of the gene (RT-PCR-Rev 5′-AATACCCGCATGCCGTTGGTGCC-3″). The reaction mix was denatured at 65°C for 5 min and reverse transcribed at 45°C for 50 min. cDNA (275 bp), the cDNA was amplified using primers Oligo b0219 *EcoRI*fw 5′-CTATGAATTCAAGAAGATATTTACGAGC-3′ and Oligo b0219 *Sph*Irv 5′-AATACCCGCATGCCGTTGGTGCC-3′, and Taq polymerase (Promega, Madison, WI, USA) according to the manufacturer's instructions. The reaction mix was amplified (denaturation at 95°C for 45 s; annealing at 58°C for 45 s; extension at 72°C for 1 min, 35 cycles). For each reverse transcriptase amplification, an additional PCR reaction on DNA-free total RNA was performed as control, in order to exclude false positive signals (Figure S3).

### Determination of minimal inhibitory concentration (MIC)

MIC was performed according to the guidelines of Clinical Laboratory Standards Institute. Pentadecanal was added directly from mother stock and solutions were prepared by two-fold serial dilutions. A total of 5 concentrations were used within the 1.6–0.1 μg/ml range. Experiments were performed in quadruplicate. The MIC was determined as the lowest concentration at which the observable bacterial growth was inhibited. No inhibition of the bacterial growth was evidenced at testedconcentrations.

## Biofilm formation of staphylococci

### Static biofilm assay

The quantification of *in vitro* biofilm production was based on the method described by Christensen with slight modifications (Artini et al., 2013; Papa et al., 2013a). Briefly, the wells of a sterile 48-well flat-bottomed polystyrene plate were filled with 400 μl of BHI or TSB medium. 1/100 Dilution of overnight bacterial cultures was added into each well. The first row contained the untreated bacteria, while each of the remaining rows contained serial dilutions of the supernatant (SN) starting from 1:2. The plates were incubated aerobically for 24 h at 37°C.

The biofilm formation was measured using crystal violet staining. After treatment, planktonic cells were gently removed; each well was washed three times with PBS and patted dry with a piece of paper towel in an inverted position. To quantify the biofilm formation, each well was stained with 0.1% crystal violet and incubated for 15 min at room temperature, rinsed twice with double-distilled water, and thoroughly dried. The dye bound to adherent cells was solubilized with 20% (v/v) glacial acetic acid and 80% (v/v) ethanol. After 30 min of incubation at room temperature, (OD) was measured at 590 nm to quantify the total biomass of biofilm formed in each well. Each data point is composed of three independent experiments, each performed at least in 3-replicates.

### Dynamic biofilm assay

To continuously monitor the biofilm development in dynamic condition, we utilized a BioFlux 2,000 microfluidic system (Fluxion Biosciences Inc., San Francisco, CA), which allows the acquisition of microscopic images over time using the experimental protocol previously set up (Iebba et al., 2014). Each flow channel connects to an input well (inlet) and an output well (outlet) on the plate. To grow the biofilm in the BioFlux system, the channels were first primed. We have filled the outlet with 100 μl of sterile distilled water and set the flow at a share setting of 1 dyne/cm^2^ for 2 min. Coating with 100 μl of 10 μg/ml fibronectin was made for 2 min at 1 dyne/cm^2^. The fibronectin binding was performed for 30 min without flow. After priming, fibronectin was aspirated from the output wells and replaced with 100 μl of fresh overnight cultures diluted to an (OD)_600_ of 0.8. The channels were seeded by pumping from the output wells to the input wells at 2.0 dyne/cm^2^ for 4 sec. Bacterial adhesion was performed for 30 min at 37°C without flow. 2.0 mL of BHI was added to the input well and pumped at 1 dyne/cm^2^ for 12 h. We used two inlet wells; in the first well we added only BHI. In the second well we added pentadecanal at a concentration of 120 μg/ml. Bright-field images were taken at 40X magnification at 1-min intervals for a total of 720 time points.

### Anti-biofilm molecule purification and identification

As reported in the previous paper (Parrilli et al., 2016), *P. haloplanktis* TAC125 supernatant deriving from sessile growth was dialyzed, and the permeated water (2 g) was then purified on a gel-filtration column (Biorad, Biogel P-2) eluted with MilliQ water. The active fractions were collected and fractionated on a C_18_ reverse phase column (Sigma, 30 mL, 40 × 0.5 cm, fraction volume 4 mL), eluted with CH_3_CN:H_2_O ranging from 10 to 95% of CH_3_CN. The fraction resulted to be active (**S**) was analyzed on a Agilent Technologies gas chromatograph 6850A equipped with a mass selective detector 5973N and a Zebron ZB-5 capillary column (Phenomenex, 30 m × 0.25 mmi.d., flow rate 1 cm^3^/min, He as carrier gas), by using the following temperature program: 150°C for 3 min, from 150 to 300°C at 15°C/min, at 300°C for 5 min.

### NMR spectroscopy

NMR spectra were performed by using a Bruker Avance-DRX 600 MHz spectrometer equipped with a cryoprobe. ^1^H-^1^H DQF-COSY, ^1^H-^1^H TOCSY, ^1^H-^13^C DEPT-HSQC, and ^1^H-^13^C HMBC experiments were recorded at 298 K in CDCl_3_. The mixing time for TOCSY experiment was 100 ms.

### Fatty aldehydes chemical synthesis

C14-C16 aldheydes were purchased from TCI. All the other tested compounds were synthesized starting from the corresponding alcohols (Sigma). 1- heptadecanol (30 mg, 0.12 mmol) was charged into a 10 mL round-bottom flask equipped with a magnetic stir bar. The solid was then dissolved in toluene (2 mL) at 20°C, and an aqueous solution of sodium bicarbonate (0.2 g, 2.38 mmol in 2 mL of deionized water) was prepared and charged into the toluene slurry. Solid iodine (2.0 eq., 60 mg, 0.24 mmol) was then charged to the alcohol followed by solid TEMPO (0.1 eq., 1.87 mg, 12.0 μmol; Miller and Hoerrner, 2003). The reaction mixture was then left under stirring for 16 h at 20°C. The batch was cooled to 5°C, diluted with ethyl acetate (2 mL), and quenched at 5°C by adding an aqueous solution of sodium sulfite 10% (312 mg sodium sulfite in 2 mL of deionized water). The quenched reaction mixture was transferred into a separatory funnel, rinsed with additional ethyl acetate (10 mL) and deionized water (10 mL), and the aqueous layer was cut away. The organic layer was then washed with 10 mL of saturated aqueous sodium bicarbonate, followed by 10 mL of brine. The organic layer was then dried over sodium sulfate, filtrated, and concentrated in vacuum to a volume of 4 mL. Then, the solution was completely dried under a stream of argon, to give the aldehyde with a 95% yield.

The same procedure was then utilized for the alcohols 1-octadecanol, 1-nonadecanol, and 1-eicosanol for the obtainment of the corresponding aldehydes.

### Statistics and reproducibility of results

The data reported were statistically validated using the Student's *t*-test comparing the mean absorbance of treated and untreated samples. The significance of differences between the mean absorbance values was calculated using a two-tailed Student's *t*-test. A *p* < 0.05 was considered significant.

### Bacterial viability and biofilm thickness determined by confocal laser scanning microscopy

For confocal microscopy biofilms were formed on Nunc™ Lab-Tek® 8-well Chamber Slides (n° 177445; Thermo Scientific, Ottawa, ON, Canada). Briefly, overnight cultures of *S. epidermidis* O-47 and RP62A grown in BHI were diluted and inoculated into each well of the chamber slide to a cell concentration of about 0.001 (OD)_600nm_. The bacterial cultures were incubated at 37°C for 20 h in presence of pentadecanal (100 μg/ml) in order to assess its anti-biofilm activity and its influence on cell viability.

The biofilm cell viability was determined with the FilmTracer™ LIVE/DEAD® Biofilm Viability Kit (Molecular Probes, Invitrogen, Carlsbad, California) following the manufacturer's instructions. After rinsing with filter-sterilized PBS, each well of the chamber slide were filled with 300 μl of working solution of fluorescent stains, containing the SYTO® 9 green fluorescent nucleic acid stain (10 μM) and Propidium iodide, the red-fluorescent nucleic acid stain (60 μM), and incubated for 20–30 min at room temperature, protected from light. All excess stain was removed by rinsing gently with filter-sterilized PBS.

All microscopic observations and image acquisitions were performed with a confocal laser scanning microscope (CLSM; LSM700-Zeiss, Germany) equipped with an Ar laser (488 nm), and a He-Ne laser (555 nm). Images were obtained using a 20X/0.8 objective. The excitation/emission maxima for these dyes are ~480/500 nm for SYTO® 9 stain and 490/635 nm for propidium iodide. Z-stacks were obtained by driving the microscope to a point just out of focus on both the top and bottom of the biofilms. Images were recorded as a series of.tif files with a file-depth of 16 bits. For each condition, two independent biofilm samples were used.

## Results

### Anti-biofilm production and purification

**Figure 1:**
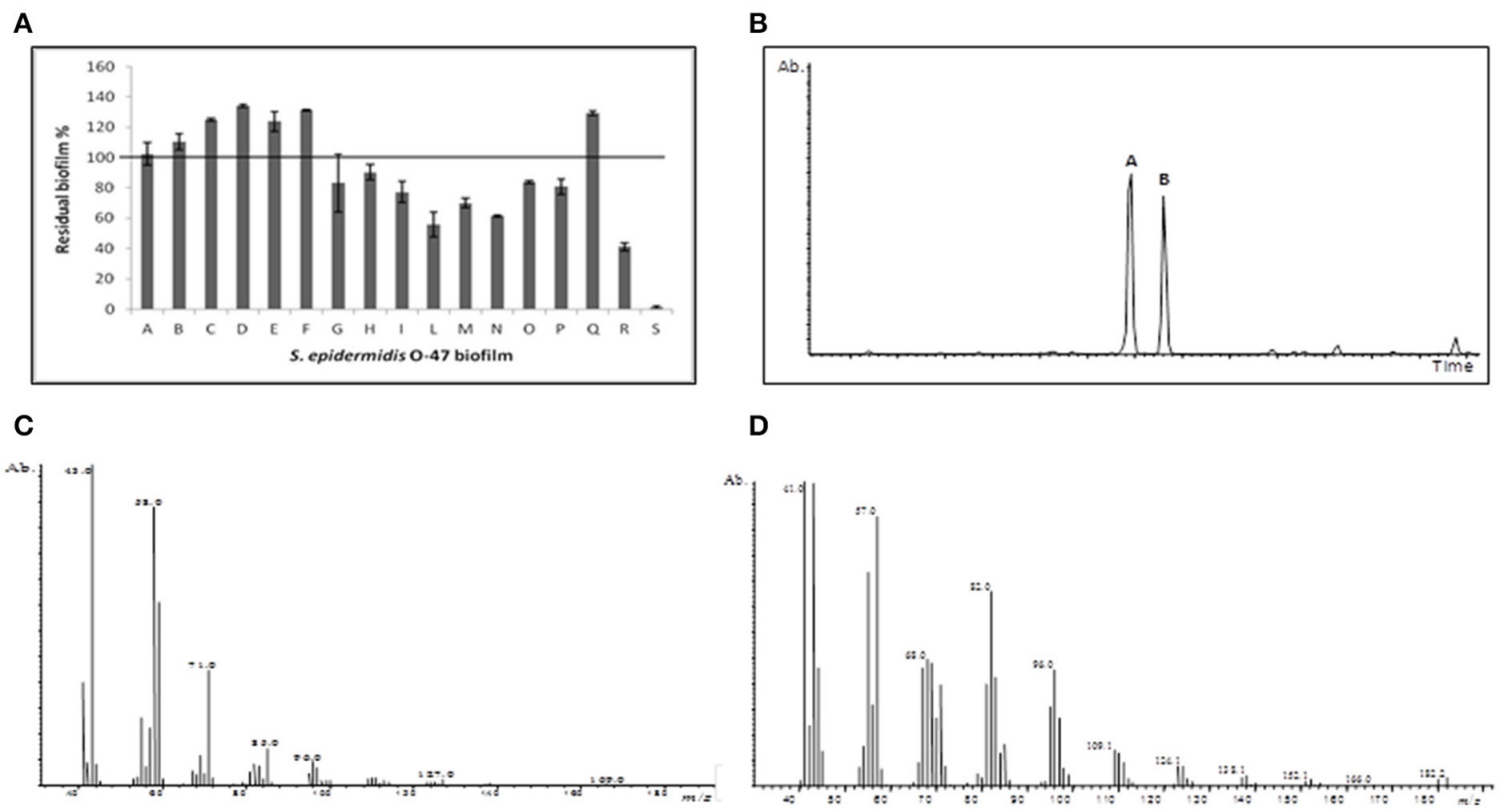
**Anti-biofilm assay and identification by GC-MS analysis. (A)** The anti-biofilm activity of different fractions obtained from reverse phase C_18_ column. The fraction S eluted with 95% acetonitrile showed the highest inhibition activity. **(B)** GC-MS chromatogram of the fraction S. **(C)** Mass spectrum of compound A, and **(D)** compound B.

We have previously (Papa et al., 2013b; Parrilli et al., 2015) demonstrated that the cell-free supernatant of the Antarctic bacterium *P. haloplanktis* TAC125 inhibits *S. epidermidis* biofilm formation and that the bacterium produces the active molecule only when it is grown in a sessile condition (Papa et al., 2013b). To obtain a sufficient amount of cell-free supernatant, we adopted a recently described (Parrilli et al., 2016) biofilm cultivation strategy for *P. haloplanktis* TAC125. This consists in a fluidized-bed reactor fermentation in the presence of floating polystyrene supports used to increase the biofilm formation (Parrilli et al., 2016). In this work the Antarctic bacterium has been grown in the presence of floating polystyrene supports in BHI in a 3 L tank reactor at 4°C without stirring, using an airflow of 6 L h^−1^ (see materials and Methods Section). The obtained cell-free supernatant was recovered after 96 h and separated from the floating polystyrene supports and the cells by a centrifugation at 13,000 rpm. Subsequently the supernatant was sterilized by filtration through membranes with a pore diameter of 0.22 μm, and then underwent dialysis treatment using a semipermeable membrane with a cut-off of 3.5 KDa. The permeate product after lyophilization was fractionated on a Biogel P-2 column and the anti-biofilm activity of each fraction was evaluated (data not shown). The fraction endowed with anti-biofilm activity on *S. epidermidis* O-47 was further purified on a C_18_ reverse phase column eluted with acetonitrile/water. Several fractions (A–S) were collected, and the anti-biofilm activity of each fraction was tested as reported in Figure 1A. Fraction S, eluted with 95% acetonitrile, showed the highest inhibitory activity on *S. epidermidis* biofilm. The analysis of the ^1^H NMR spectrum of this fraction revealed a signal at δ 9.77 ppm, which immediately suggested the presence of an aldehyde (MOLBASE.COM CAS. No. 2765-11-9). This was believed to be aliphatic, due to the presence of intense signals in the range between δ 0.1–2.5 ppm (data not shown). To confirm this hypothesis, and to check the purity of the sample, a GC-MS analysis was performed (Figure 1B). The chromatogram clearly indicated two different compounds, named **A** and **B**, the EI mass spectra of which are reported (Figures 1C,D). Both spectra showed signals at *m/z* 43, 57, 71, and 85, that are typical of a straight chain hydrocarbon backbone, thus confirming the aliphatic character of the two molecules. As this type of fragmentation can be attributed to a broad range of compounds, a research in the NIST library was required. The results indicated with a high score a 2-tridecanone and a pentadecanal for **A** and **B**, respectively. As the inactive fraction Q mainly contains the ketone whereas fraction S shows a higher content of the pentadecanal, we concluded that the activity should be assigned to the aldehyde. To exclude the possibility that the mass spectrum of **B** could belong to an aldehyde with a short difference in the carbon chain length, we injected into the gas chromatograph commercial C14-C16 aldehydes. The retention time of compound **B** proved to correspond to that of pentadecanal.

**Figure 2:**
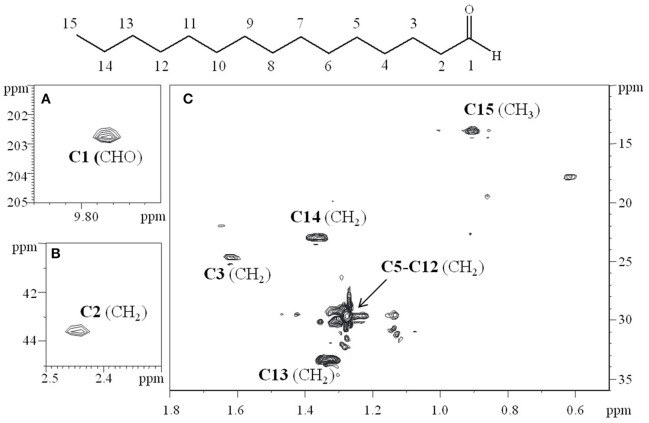
**Relevant sections of ^**1**^H-^**13**^C HSQC spectrum, recorded in CDCl_**3**_ at 298K at 600 MHz. (A)** The correlation at δ 9.79/203.1 ppm, clearly indicated the aldehydic functional group of the molecule. **(B)** The carbon signal at δ 203.1 ppm is in turn correlated in the HMBC experiment (data not shown), with the protons at δ 2.43 ppm (CH_2_ C2). **(C)** The attribution of the aliphatic chain (C3–C15).

To confirm the data obtained from GC-MS, the **S** fraction was analyzed by 2D-NMR spectroscopy. In particular, two-dimensional ^1^H-^1^H DQF-COSY (double quantum-filtered correlation spectroscopy), ^1^H-^1^H TOCSY (total correlation spectroscopy), ^1^H-^13^C DEPT-HSQC (distortionless enhancement by polarization transfer-heteronuclear single quantum coherence), and ^1^H-^13^C HMBC (heteronuclear multiple bond correlation) experiments were performed. The proton at δ 9.79 ppm, that clearly indicated an aldehyde functional group on the molecule, displayed a correlation with a carbon at δ 203.1 ppm (Figure 2A; data not shown). In turn, this carbon showed a long range scalar connectivity with protons at δ 2.43 ppm (CH_2_,C2; Figure 2B). Starting from this proton signal, the correlations revealed in the COSY and TOCSY spectra allowed us to attribute all the CH_2_(C3-C14) that constitute the aliphatic chain. The last signal, attributable to the CH_2_C14 (δH/C 1.36/22.9), showed a long-range cross-peak, with the methyl group at δH/C 0.9/13.6 ppm (Figure 2C).

**Figure 3:**
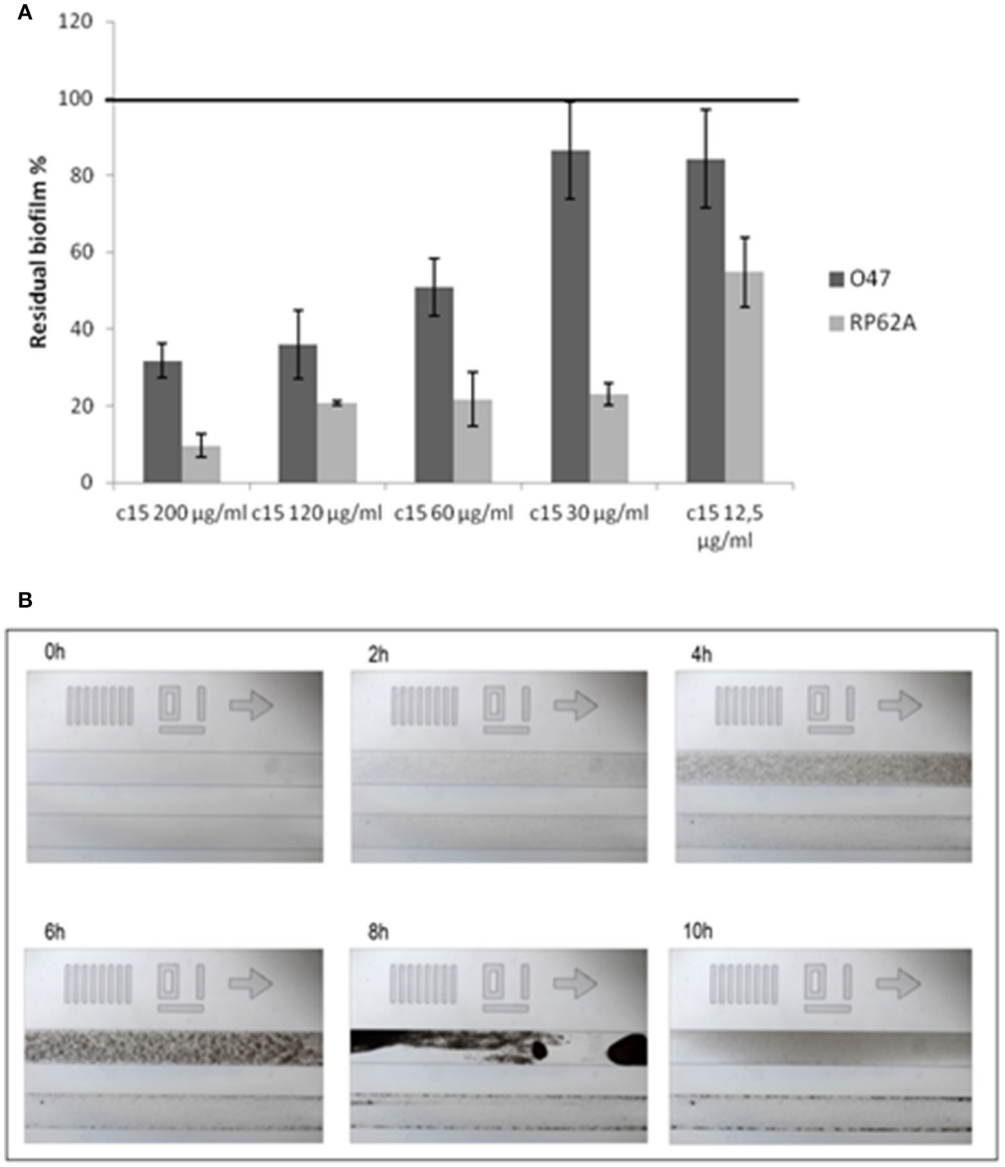
**Pentadecanal anti-biofilm activity on ***S. epidermidis*** biofilm formation**. **(A)** The effect of pentadecanal at different concentrations on biofilm formation of *S. epidermidis* O-47 and *S. epidermidis* RP62A. The data are reported as percentages of residual biofilm. Each data point is composed of three independent samples. **(B)** Biofilm formation of *S. epidermidis* O-47 in a BioFlux system in the presence of pentadecanal. Each image contains two channels: the bottom channel is the pentadecanal-treated sample and the top channel is the control. Bright-field microscopic images were collected at 1-min intervals. The images presented were taken from the complete set of 720 images taken at 40x magnification.

A confirmation of the correct assignment of the pentadecanal structure was obtained by testing the commercial product on *S. epidermidis* biofilm, that was found to be active (Figure 3A). Consequently, commercial pentadecanal was used for all further experiments.

### Anti-biofilm activity and specificity of pentadecanal on *S. epidermidis* biofilm

Once the structure of the anti-biofilm molecule had been identified, the first step was to confirm the absence of any antimicrobial activity of pentadecanal on the staphylococcal reference strains by determining the minimum inhibitory concentration. The data obtained indicated no bacteriostatic and no bactericidal activity within the range of concentrations explored (the highest concentration explored was 1,6 mg/ml). Preliminary experiments were also carried out to assess the effects of the molecule on the planktonic growth rate of *S. epidermidis*. The results obtained showed that pentadecanal did not affect the staphylococcal duplication rate. Bacterial growth curves were superimposable both in the presence and absence of pentadecanal (data not shown).

The pentadecanal activity on biofilm formation was assessed on two different *S. epidermidis* strains (Figure 3A), according to previously reported data that demonstrated the cell-free supernatant activity of *P. haloplanktis*TAC125 against the *S. epidermidis* O-47 and *S. epidermidis* RP62A biofilms (Papa et al., 2013b). As expected, the pentadecanal inhibitory effect was clearly dose-dependent with an efficacy higher than 50% already at 60 μg/ml (50.9% residual biofilm for *S. epidermidis* O-47 and 21.8% for *S. epidermidis* RP62A, respectively). All previously described experiments were performed in BHI medium. The pentadecanal anti-biofilm activity was also tested in TSB medium to verify its effectiveness in function of growth medium composition. The results demonstrated that this latter did not influence the anti-biofilm ability of pentadecanal (untreated bacteria 0.761 ± 0.137; treated bacteria 0.152 ± 0.020).

The pentadecanal anti-biofilm efficacy was also tested in dynamic conditions with the BioFlux system, a microfluidic device that precisely controls the flow of growth medium between two interconnected wells of a microtiter plate. By positioning the channel connecting the two wells over a window accessible for viewing by microscopy, biofilm growth can be monitored in a time-course assay in which images are collected at 1-min intervals. In Figure 3B selected images of the BioFlux analysis are reported, showing the biofilm development of *S. epidermidis* O-47 at different times, in the absence or in the presence of pentadecanal (top and bottom lanes of each panel, respectively). The bacteria were seeded in both channels visible in each frame and after 30 min the flow was applied. The images collected showed an initial rapid growth of the bacteria, resulting in a confluent “lawn” of cells that was followed by a period of detachment. Pentadecanal clearly impaired the biofilm formation confirming the results obtained in the static system.

**Figure 4:**
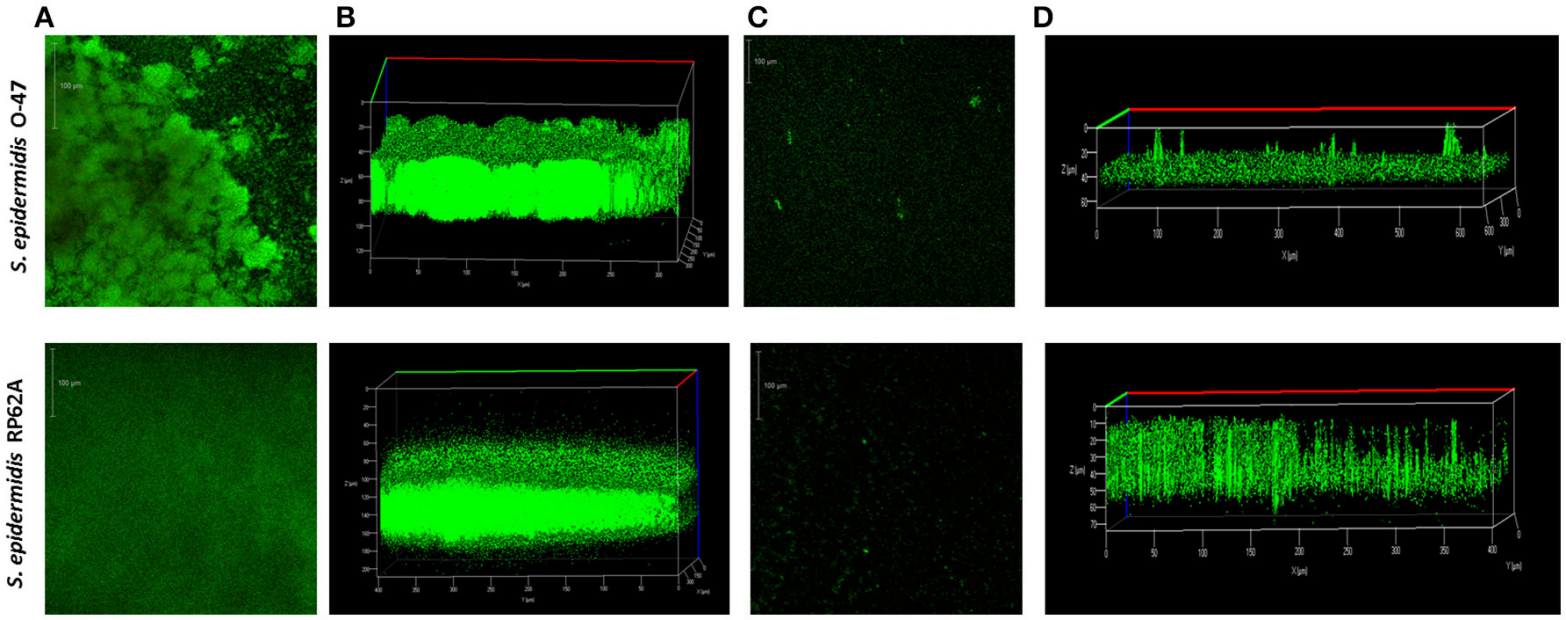
**CLSM of ***S. epidermidis*** O-47 and ***S. epidermidis*** RP62A biofilms in the presence and absence of pentadecanal**. **(A)** CLSM of *S. epidermidis* O-47 and *S. epidermidis* RP62A biofilms in BHI medium without pentadecanal and **(C)** with pentadecanal 100 μg/ml. The bacteria were grown in chamber slides for 20 h and then stained with LIVE/DEAD reagents. The green fluorescence (SYTO9) indicates viable cells PI and the red fluorescence (PI) indicates dead cells. **(B)** Z-stack analysis of *S. epidermidis* O-47 and RP62A biofilms without pentadecanal. **(D)** Z-stack analysis of *S. epidermidis* O-47 and RP62A biofilms treated with 100 μg/ml pentadecanal.

The pentadecanal effect on *S. epidermidis* O-47 biofilm was further investigated by confocal laser scanning microscopy (Figures 4A–D). CLSM was used to analyse the biofilm structure and viability, as shown in Figures 4B,D. In the presence of pentadecanal, as expected, the biofilm thickness decreased significantly with a notable alteration in the architecture. This effect was evident both on *S. epidermidis* O-47 biofilm and on *S. epidermidis* RP62A biofilm. Moreover, the viability of cells encapsulated in the biofilm in the presence and in the absence of pentadecanal was evaluated by live/dead staining. As shown in Figure 4C, cells exposed to pentadecanal were alive (green indicates viable cells while red indicates dead cells) confirming that the long-chain fatty aldehyde had no bactericidal activity on the *S. epidermidis* cells living in the biofilm.

**Figure 5:**
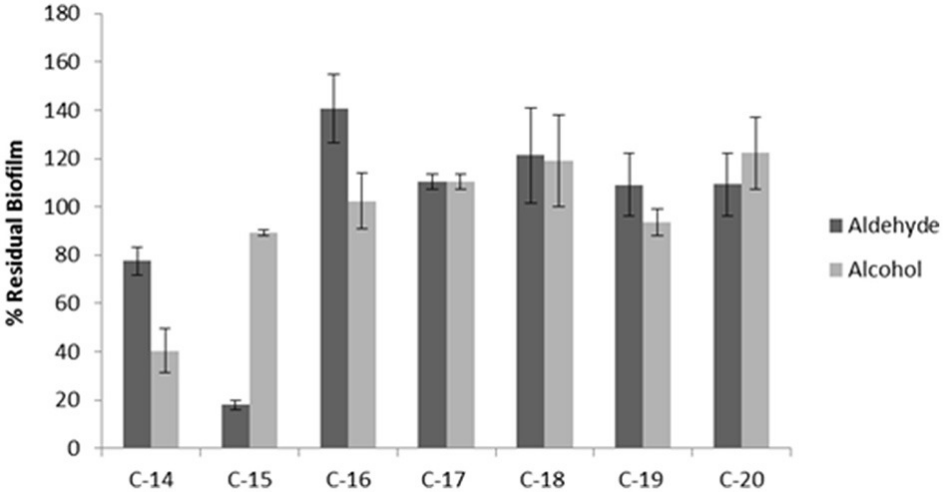
**Anti-biofilm activity of different aldehydes and alcohols on ***S. epidermidis*** O-47**. The anti-biofilm activity of different long-chain aldehydes and alcohols on *S. epidermidis* O-47. The data are reported as percentages of residual biofilm. Each data point is composed of four independent samples.

In order to investigate the chemical features (the length of the chain and the functional group nature) that are essential for the anti-biofilm activity, the effect of similar aldehydes and corresponding alcohols, characterized by different lengths of the aliphatic chain in the range from C-14 to C-20, were analyzed and the results are summarized in Figure 5. The data are reported as percentages of residual biofilm after the treatment in comparison with the untreated biofilm of *S. epidermidis* O-47. Our results clearly showed that, except for a partial reduction observed after treatment with the alcohol at the C-14 chain, the anti-biofilm activity was limited to pentadecanal.

### Pentadecanal as an AI-2 signaling molecule in the LuxS/AI-2 QS system

**Figure 6:**
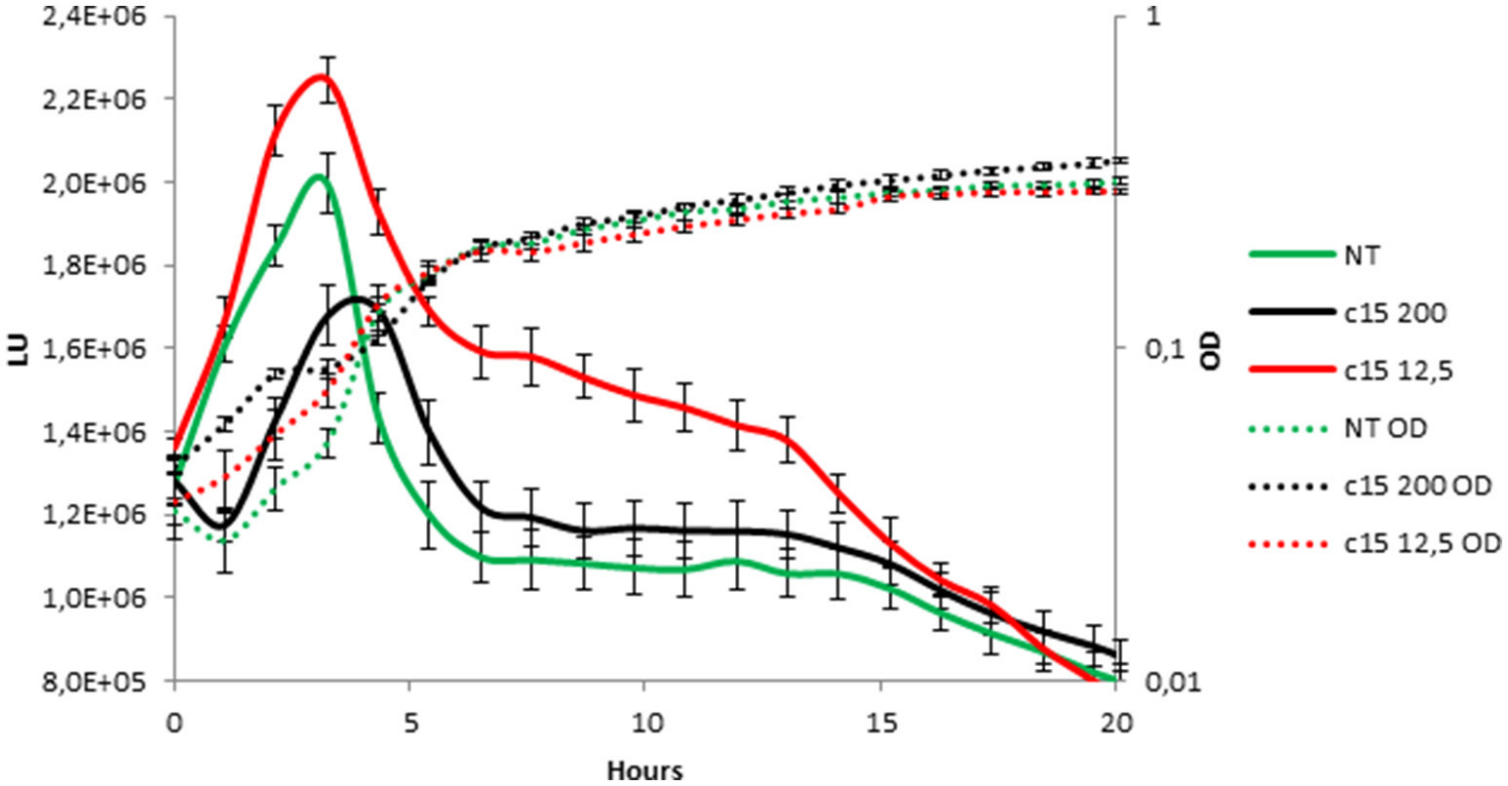
*****V. harveyi*** LuxS/AI-2 QS system responds to the presence of pentadecanal**. Bioluminescence (solid lines) and growth curves (dotted lines) of the *V. harveyi* BB170 strain incubated for 20 h in the presence of only the AB medium (green lines), 12.5 μg/mL pentadecanal (black lines) and 200 μg/mL pentadecanal concentrations (red lines).

To test whether the anti-biofilm activity displayed by pentadecanal is correlated with a modulation/activation of the AI-2 quorum sensing system, the effect of pentadecanal was investigated on a *V. harveyi* BB170 strain (luxN::tn5Kan), a mutant sensor strain that responds only to AI-2 autoinducers. The *V. harveyi* BB170 bioluminescence was monitored (Figure 6) after adding two different concentrations of pentadecanal. At a low concentration (12.5 μg/ml) the pentadecanal was able to increase the *V. harveyi* bioluminescence (Figure 6), indicating that the *V. harveyi* LuxS/AI-2 QS system responds to pentadecanal. At a higher concentration (200 μg/ml) pentadecanal reduced the bioluminescence (Figure 6). In this condition, pentadecanal probably inhibits the *V. harveyi* luciferase. Since the *V. harveyi* luciferase physiological substrate is tetradecanal (Ulitzur and Hastings, 1979), the bioluminescence in *V. harveyi* BB170 was also monitored after adding two different concentrations of tetradecanal (Figure S1). As expected, a higher concentration (200 μg/ml) of tetradecanal reduced the *V. harveyi* bioluminescence due to the luciferase substrate inhibition, at low concentrations tetradecanal had no effect on the *V. harveyi* bioluminescence (Figure S1).

### The role of pentadecanal in the *P. haloplanktis*TAC125 physiology

To assess the role of pentadecanal in the *P. haloplanktis*TAC125 physiology, we investigated the conditions necessary for pentadecanal production and the possible synthetic pathway involved.

The *in silico* analysis of the *P. haloplanktis*TAC125 genome (EMBL under accession nos. CR954246 and CR954247). did not allow us to identify the genes coding for enzymes known to be able to synthesize fatty aldehydes, like fatty acid reductase (EC 1.2.1.50 (Gahan, 2012) or fatty acid peroxidase (EC1.11.1.3, Martin and Stumpf, 1959). However, the analysis allowed the identification of a gene (PSHAb0219) coding for an aldehyde dehydrogenase B (EC1.2.1.3), an enzyme annotated as a reversible aldehyde oxidoreductase NAD(P)^+^-dependent with a wide specificity (http://www.genoscope.cns.fr/agc/microscope/mage/viewer.php). The aldehyde oxidoreductase, in specific conditions, could be able to produce pentadecanal by reducing the corresponding fatty acid. To assess if this was a correct hypothesis, we first investigated the presence of the pentadecanoic acid in *P. haloplanktis* TAC125 cells. A GC-MS analysis of fatty acid methyl esters from *P. haloplanktis* TAC 125 cells, grown either in sessile or in planktonic conditions, revealed the presence of pentadecanoic acid (Figure S2).

Once the presence of the possible substrate had been verified, both in sessile and in planktonic conditions, a RT PCR analysis of the PSHAb0219 gene was performed to assess if the gene transcription is growth condition-dependent. *P. haloplanktis* TAC125 cells were cultured in planktonic or in sessile conditions and total RNA was extracted from the two different samples. The PSHAb0219 gene amplification was obtained with specific oligonucleotide pairs (see Material and Methods Section). The results of the RT-PCR experiments (Figure S3) showed that a PSHAb0219 gene transcription was clearly detected in the RNA samples extracted from *P. haloplanktis* TAC125 cells grown in both conditions. Several experiments were then attempted to delete the PSHAb0219 gene by using genetic tools for the creation of insertion/deletion mutants already available (Parrilli et al., 2010). However, these experiments were not successful probably due to the fact that PSHAb0219, in tested conditions, is likely to be an essential gene.

**Figure 7:**
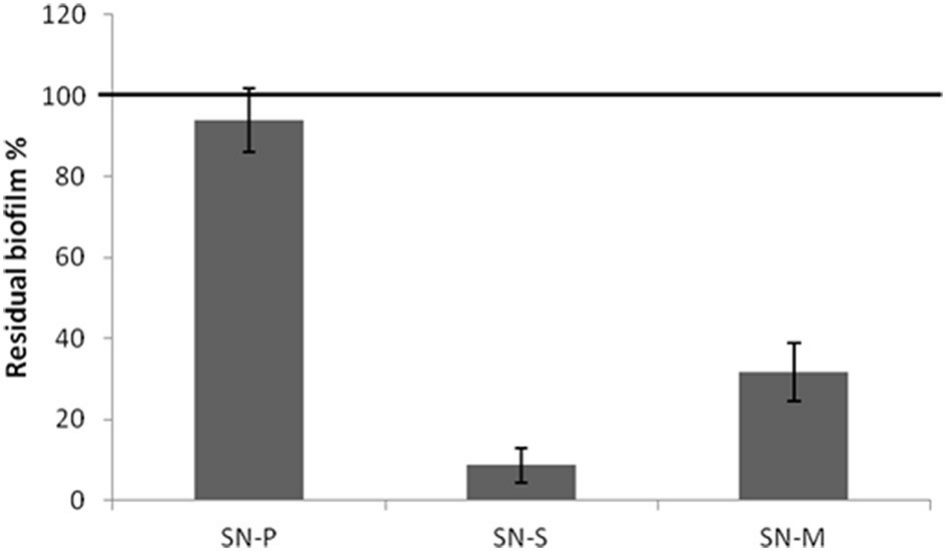
**The cell-free supernatant of ***P. haloplanktis*** TAC125 grown in microaerobiosis has an anti-biofilm activity** *S. epidermidis* O-47 biofilm formation after incubation with *P. haloplanktis* TAC125 cell-free supernatants obtained from sessile (SN-S) and planktonic growths (SN-P) and from microaerobiosis growth (SN-M). The data are reported as percentages of residual biofilm. Each data point represents the mean ± SD of at least three independent samples.

Since the pentadecanoic acid was produced in both sessile and planktonic conditions and the PSHAb0219 gene was transcribed in both settings, a possible explanation of the presence of pentadecanal only in the supernatant of the sessile form could be that the aldehyde oxidoreductase catalyses the reduction of the acid to aldehyde only in the biofilm condition. In particular, in oxygen limitation, a condition occurring in biofilm (Wessel et al., 2014), bacterial cells could reduce the fatty acids in order to obtain the oxidized cofactor (NAD(P)^+^) necessary for cell metabolism. To verify this hypothesis *P. haloplanktis* TAC125 cells were grown, in an automatic bioreactor, at 4°C in microaerobiosis under agitation, a condition where the measured oxygen pressure is kept constantly below 5% saturation (measured oxygen pressure <5%)(see Materials and Methods Section). The anti-biofilm activity of the cell-free supernatant of *P. haloplanktis* TAC125 grown in microaerobiosis (SN-M) was tested on *S. epidermidis* biofilm. The SN-M proved to be able to inhibit the *S. epidermidis* O-47 biofilm formation (Figure 7), thus indicating that the pentadecanal production occurs also in microaerobiosis. This experiment demonstrates that oxygen availability is a crucial parameter for pentadecanal synthesis and proves to be the main difference between sessile and planktonic conditions, so determining the anti-biofilm production.

Although pentadecanal could be not defined as a strictly biofilm-specific metabolite, its effect on *P. haloplanktis* TAC125 biofilm formation and development was tested. The aldehyde presence proved to have no effect (Figure S4) on the Antarctic bacterium biofilm development. Moreover, pentadecanal proved to be inactive against the mature biofilm of *P. haloplanktis* TAC125 (data not shown).

## Discussion

The emergence of *Staphylococcus epidermidis* as an opportunistic pathogen is closely related to the biofilm forming capability of this bacterial species, especially during the biofilm-associated infection of indwelling medical devices. The increasing use of implanted medical devices heightens the importance of *S. epidermidis* as a human pathogen. Our research has been aimed at discovering anti-biofilm molecules from natural sources, such as marine microbiota, since we are convinced that this approach will lead to the discovery of novel and unforeseen compounds. Cold-adapted marine bacteria represent a still underexploited source of biodiversity able to synthesize a broad range of bioactive compounds, including anti-biofilm molecules. Previous results (Papa et al., 2013b; Parrilli et al., 2015) have demonstrated that the culture supernatant of Antarctic marine bacterium *P. haloplanktis* TAC125 impairs the formation of *S. epidermidis* biofilm.

In this paper we applied a *P. haloplanktis* TAC125 biofilm cultivation strategy in automatic bioreactor (Parrilli et al., 2016) and an efficient activity-guided purification protocol to produce and purify the anti-biofilm molecule. The structure of the anti-biofilm molecule, obtained by NMR and mass spectrometry, corresponds to pentadecanal, a long-chain fatty aldehyde.

The pentadecanal activity on biofilm formation was confirmed on two different *S. epidermidis* strains in static condition and, by the BioFlux system, also in dynamic conditions. BioFlux can reproduce environmental or physiological conditions by precisely controlling shear flow, bridging the gap between *in vitro* and *in vivo* assays. In Bioflux conditions bacteria progress through a series of developmental steps, ultimately forming a multicellular structure containing differentiated cell populations. The observation of the growing biofilm at various time-points throughout this process provides a glimpse of the temporal changes that occur. Flow biofilm is more closely related to natural biofilms and can differ from static biofilms in terms of the hydrodynamic influences on cell signaling. The finding that pentadecanal is active also in dynamic conditions demonstrates that this molecule shows great promise in terms of its use *in vivo* systems.

It is interesting to note that most of the known anti-biofilm molecules also display an antibacterial activity (bactericidal or bacteriostatic). In contrast, pentadecanal lacks any antibacterial activity against free-living bacteria and against bacteria entrapped in biofilm matrix. Indeed as demonstrated by CLSM analysis the presence of pentadecanal reduces the biofilm thickness without affecting the viability of *S. epidermidis* cells living in the biofilm. Therefore, pentadecanal targets the adhesive properties without affecting the bacterial viability, a type of behavior which should prevent the development of escape mutants. This property makes pentadecanal particularly interesting and suggests its possible use in combination with conventional antibiotic therapy.

The specificity of pentadecanal action on *S. epidermidis* biofilm has been demonstrated by testing chemical analogs differing either in the length of the aliphatic chain or in their functional group properties. The results reported demonstrate that both the length of the aliphatic chain and the functional group properties are essential for the pentadecanal activity against *S. epidermidis* biofilm.

With regard to the anti-biofilm mode of action, several observations prompted us to explore the idea that the action of pentadecanal on *S. epidermidis* biofilm could be related to its possible interference in the quorum sensing system. The most important and best-characterized quorum-sensing system in staphylococci is the accessory gene regulator (*agr*) system. Remarkably, the *P. haloplanktis* TAC125 anti-biofilm molecule is active on the clinical isolate O-47, which is a naturally occurring *agr* mutant (Vuong et al., 2003); therefore a possible effect mediated by quorum sensing should be directed at the LuxS/AI-2 quorum sensing system (Xu et al., 2006; Li et al., 2008), the second quorum sensing system present in *S. epidermidis*. The role of the LuxS/AI-2 QS system in *S. epidermidis* biofilm regulation is under debate. Xu and co-authors have suggested that the autoinducer 2 (AI-2) negatively regulates the expression of the *ica* gene [*ica* operon encodes enzymes responsible for the production of polysaccharide intercellular adhesion PIA (Otto, 2008)] at the transcriptional level, reducing the PIA synthesis and biofilm formation (Xu et al., 2006). In contrast, a recent paper of Xue et al. (2015) has reported that AI-2 increased biofilm formation in *S. epidermidis* RP62A by enhancing the transcription of the *ica* operon. Moreover, in a paper (Li et al., 2008) concerning AI-2 dependent gene regulation in *S. epidermidis*, it has been demonstrated that AI-2 controls also the levels of phenol-soluble modulins (PSMs), key molecules able to lead to biofilm detachment of *S. epidermidis* biofilm *in vivo* e *in vitro* (Otto, 2013). In any case, the crucial role of the LuxS/AI-2 QS system in *S. epidermidis* biofilm formation is evident, while its role in *Staphylococcus aureus* biofilm formation is less clear; indeed, several studies suggest that the LuxS/AI-2 quorum sensing system has no role in *S. aureus* biofilm formation (Doherty et al., 2006; Cluzel et al., 2010). The differences in the involvement of AI-2 molecules in biofilm formation between *S. aureus* and *S. epidermidis* could explain why the *P. haloplanktis* TAC125 anti-biofilm compound is effective against the *S. epidermidis* biofilm but is not able to inhibit the *S. aureus* biofilm (Papa et al., 2013b). To test whether the anti-biofilm activity displayed by pentadecanal is correlated with a modulation/activation of the AI-2 quorum sensing system, the effect of pentadecanal was investigated on a *V. harveyi* mutant sensor strain that responds only to the AI-2 autoinducers.

The results reported demonstrate that at low concentration the pentadecanal is able to increase the *V. harveyi* bioluminescence, indicating that the *V. harveyi* LuxS/AI-2 QS system responds to pentadecanal, at a higher concentration it reduces the bioluminescence. In this condition, pentadecanal probably inhibits the *V. harveyi* luciferase. The key reaction in bacterial bioluminescence is the oxidation of the luciferase-catalyzed FMNH_2_ and the long chain aliphatic aldehyde (Byers et al., 1988), and, consequently, the bacterial luciferase, that can utilize fatty aldehydes of varying chain lengths, is inhibited by the high levels of aldehydes (Francisco et al., 1993). Therefore, it is reasonable to assume that also pentadecanal could be an inhibitor of the *V. harveyi* luciferase. Since the *V. harveyi* luciferase physiological substrate is tetradecanal (Ulitzur and Hastings, 1979), the bioluminescence in *V. harveyi* mutant was also monitored in presence of tetradecanal, and, as expected, a higher concentration reduced the *V. harveyi* bioluminescence due to the luciferase substrate inhibition. It is interesting to note that at low concentrations tetradecanal had no effect on the *V. harveyi* bioluminescence.

The results reported demonstrate that the *V. harveyi* LuxS/AI-2 QS system responds to pentadecanal. These data suggest that the pentadecanal anti-biofilm activity in *S. epidermidis* could be due to an interference in the AI-2 quorum sensing system. Although several studies will be necessary to clarify the molecular details of the pentadecanal action on *S. epidermidis* biofilm formation, this is the first report on the action of a long-chain fatty aldehyde as an anti-biofilm molecule that works as a signaling molecule in an AI-2 QS system. Further investigations will be necessary to clarify if this modulation occurs also in the *S. epidermidis* AI-2 quorum sensing system.

Besides the characterization of pentadecanal anti-biofilm activity on *S. epidermidis* biofilm, we have investigated the metabolic role and synthesis of this long fatty compound in the Antarctic source strain. In a previous work we examined the anti-biofilm activity of cell-free supernatants obtained from the *P. haloplanktis* TAC125 grown in sessile or in planktonic conditions on different staphylococci (Papa et al., 2013b). Our results demonstrated that only when *P. haloplanktis*TAC125 is grown in sessile condition the cell-free supernatant inhibit the biofilm formation of *S. epidermidis*. This latter result was explained by considering that biofilm specific environmental conditions may induce a profound genetic and metabolic rewiring allowing the production of biofilm-specific metabolites (Beloin and Ghigo, 2005). To evaluate if pentadecanal can be considered a biofilm-specific metabolite, we investigated the conditions necessary for pentadecanal production and the possible synthetic pathway involved. The results reported strongly suggest that a *P. haloplanktis*TAC125 reversible aldehyde oxidoreductase NAD(P)+-dependent catalyses, in oxygen limitation, the reduction of the pentadecaonic acid to aldehyde to obtain the oxidized cofactor (NAD(P)+) necessary for cell metabolism. Therefore, the pentadecanal production occurs in the biofilm condition and in microaerobiosis due to the reduced oxygen availability that characterizes these two growth conditions. The oxygen availability results a crucial parameter for pentadecanal synthesis and proves to be the main difference between sessile and planktonic conditions, so determining the anti-biofilm production. Moreover, the results described in this paper indicate that the long-chain fatty aldehyde is not involved in the control/modulation of the Antarctic bacterium biofilm development.

In conclusion, this is the first report both on the action of a long chain fatty aldehyde as an anti-biofilm molecule and on an aldehyde working as a signaling molecule in the AI-2 QS system. We believe that this paper endorses the potential of cold-adapted marine bacteria as a source of bioactive compounds of interest, and contributes to the development of innovative approaches for the prevention and treatment of *S. epidermidis* biofilm-associated infections.

## Author contributions

AC: Performed the experiments, suggested critical parameters in design of experiments and co-wrote the paper. RP: Performed the experiments, suggested critical parameters in design of experiments and co-wrote the paper. AR: Performed the experiments and co-wrote the paper. FS, MZ and MT: Performed the experiments. LS: Provided advice in performance of experiments and edited paper. GM: Provided advice in performance of experiments and co-wrote paper. MC: Suggested critical parameters in design of experiments and co-wrote paper. MLT: Suggested critical parameters in design of experiments and co-wrote paper. MA: Provided advice in performance of experiments and edited paper. EP: Designed the experiments, provided advice in performance of experiments and wrote the paper.

## Funding

This work was supported by Programma Nazionale di Ricerche in Antartide 2013/B1.04 Tutino.

### Conflict of interest statement

The authors declare that the research was conducted in the absence of any commercial or financial relationships that could be construed as a potential conflict of interest.
