# Supplementary material for: Anti-Biofilm Activity of a Long-Chain Fatty Aldehyde from Antarctic Pseudoalteromonas haloplanktis TAC125 against Staphylococcus epidermidis Biofilm
Source: Front Cell Infect Microbiol. 2017 Feb 23;7:46. doi: 10.3389/fcimb.2017.00046 (PMC5322152; doi:10.3389/fcimb.2017.00046)
Supplement: Supplementary file 1 [file DataSheet1.DOC]

Supplementary Material

**Anti-biofilm activity of a long-chain fatty aldehyde from Antarctic *Pseudoalteromonas haloplanktis* TAC125 against *Staphylococcus epidermidis* biofilm**

Angela Casillo^1,§^, Rosanna Papa^2,§^, Annarita Ricciardelli^1^, Filomena Sannino^1^, Marcello Ziaco^1^, Marco Tilotta^2^, Laura Selan^2^, Gennaro Marino^1^, Maria Michela Corsaro^1^, Maria Luisa Tutino^1^, Marco Artini^2^, Ermenegilda Parrilli^1,*^

***Corresponding author:** Ermenegilda Parrilli; Tel.: +39081674474, Fax: +39081674313, erparril@unina.it

**Supporting Information**


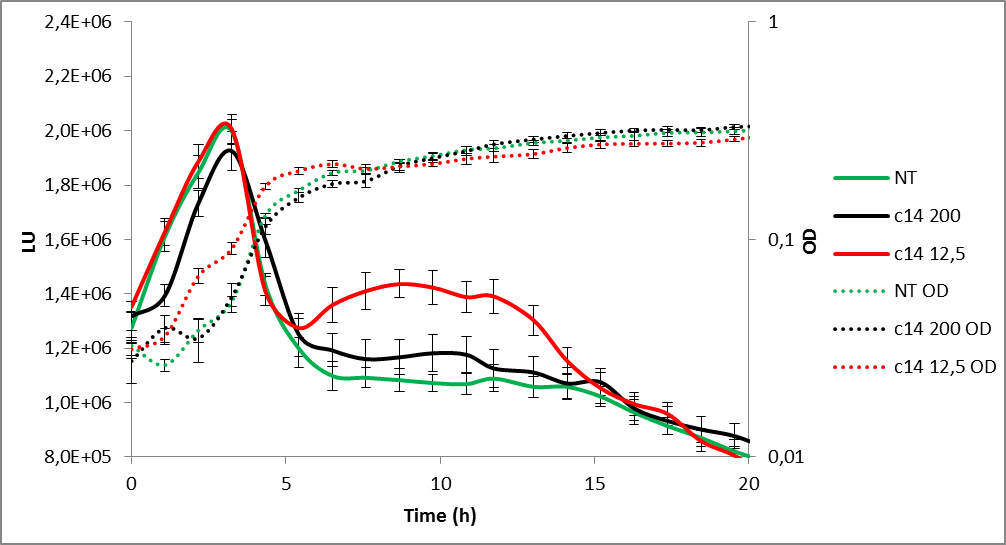


**Figure S1. *V. harveyi* bioluminescence in presence of tedradecanal**

Bioluminescence (solid lines) and growth curves (dotted lines) of *V. harveyi* BB170 strain incubated for 20 h in the presence of medium only (green lines) and in presence of 12.5µg/mL tedradecanal (black lines) and in presence of 200µg/mL tetradecanal (red lines).

**Figure S2. Fatty acids methyl esters analysis.**

(A) Fatty acids methyl esters analysis from *P. haloplankti*s TAC 125 cells grown in planktonic condition at 4°C. (B) Fatty acids methyl esters analysis from *P. haloplanktis* TAC 125 cells grown in sessile condition at 4°C.

PC

1

2

NC

Mk


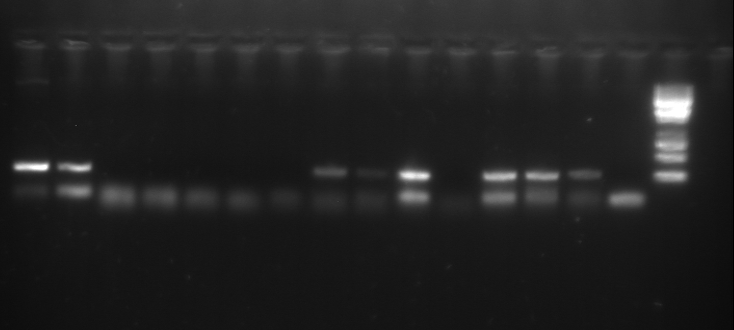

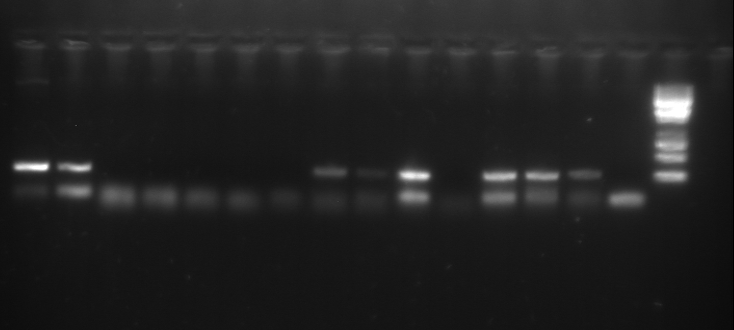

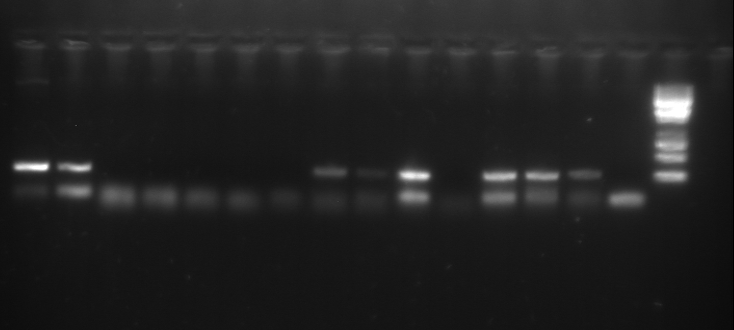

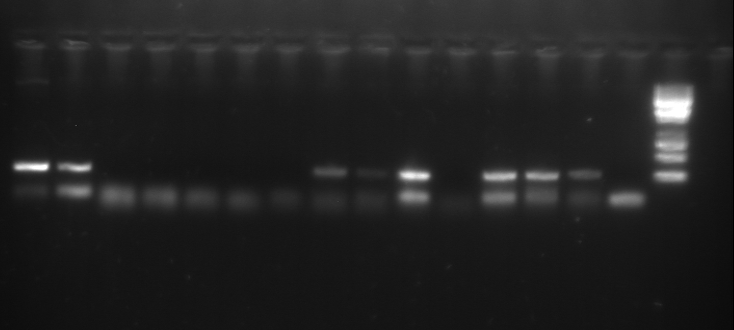


**Figure S3. Transcriptional analysis of PSHAb0219 gene**.

Total RNA samples were extracted from cells grown in planktonic (lane 1) and biofilm (lane 2). *P. haloplanktis* TAC125 genomic DNA was used as positive control (PC). To exclude the presence of artifacts due to the annealing of oligonucleotides, a PCR reaction was performed also in the absence of template (negative control, NC). Molecular marker (Mk, MBI Fermentas #SM0311).


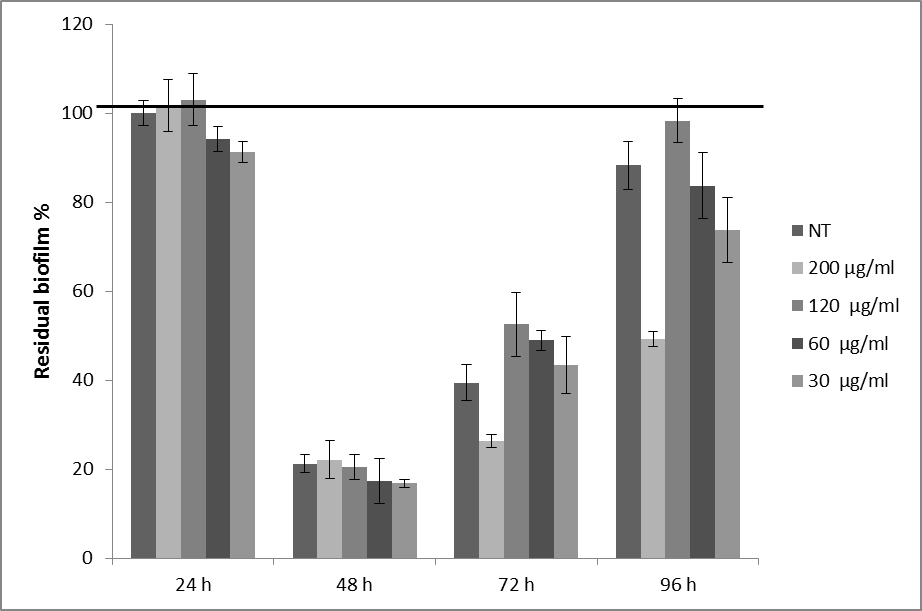


**Figure S4. Pentadecanal has no effect on *P. haloplanktis* TAC125 biofilm formation**

*P. haloplanktis* TAC125 biofilm formation in presence of different concentrations of pentadecanal (starting from 200µg/ml). Each data point represents the mean ± SD of four independent samples.
